# Dietary Total Antioxidant Capacity and Colorectal Cancer in the Italian EPIC Cohort

**DOI:** 10.1371/journal.pone.0142995

**Published:** 2015-11-13

**Authors:** Marilena Monica Vece, Claudia Agnoli, Sara Grioni, Sabina Sieri, Valeria Pala, Nicoletta Pellegrini, Graziella Frasca, Rosario Tumino, Amalia Mattiello, Salvatore Panico, Benedetta Bendinelli, Giovanna Masala, Fulvio Ricceri, Carlotta Sacerdote, Vittorio Krogh

**Affiliations:** 1 Department of Biomedical Science and Human Oncology, Aldo Moro University of Bari, Bari, Italy; 2 Epidemiology and Prevention Unit, Fondazione IRCCS Istituto Nazionale dei Tumori, Milan, Italy; 3 Department of Food Science, University of Parma, Parma, Italy; 4 Cancer Registry, Department of Prevention, ASP 7, Ragusa, Italy; 5 Department of Clinical and Experimental Medicine, University of Naples Federico II, Naples, Italy; 6 Molecular and Nutritional Epidemiology Unit, Cancer Research and Prevention Institute (ISPO), Florence, Italy; 7 Unit of Epidemiology, Regional Health Service ASL TO3, Grugliasco (TO), Italy; 8 Unit of Cancer Epidemiology, University of Turin and Center for Cancer Prevention, Turin, Italy; Robert Gordon University, UNITED KINGDOM

## Abstract

**Background:**

Colorectal cancer is the third most common cancer worldwide. Diet has been hypothesized as involved in colorectal cancer etiology, but few studies on the influence of total dietary antioxidant intake on colorectal cancer risk have been performed.

**Methods:**

We investigated the association between colorectal cancer risk and the total antioxidant capacity (TAC) of the diet, and also of intake of selected antioxidants, in 45,194 persons enrolled in 5 centers (Florence, Naples, Ragusa, Turin and Varese) of the European Prospective Investigation into Cancer and Nutrition (EPIC) Italy study. TAC was estimated by the Trolox equivalent antioxidant capacity (TEAC) assay. Hazard ratios (HRs) for developing colorectal cancer, and colon and rectal cancers separately, adjusted for confounders, were estimated for tertiles of TAC by Cox modeling, stratifying by center.

**Results:**

Four hundred thirty-six colorectal cancers were diagnosed over a mean follow-up of 11.28 years. No significant association between dietary TAC and colorectal cancer incidence was found. However for the highest category of TAC compared to the lowest, risk of developing colon cancer was lower (HR: 0.63; 95% CI: 0.44–0.89, P trend: 0.008). By contrast, increasing TAC intake was associated with significantly increasing risks of rectal cancer (2nd tertile HR: 2.09; 95%CI: 1.19–3.66; 3rd tertile 2.48 95%CI: 1.32–4.66; P trend 0.007). Intakes of vitamin C, vitamin E, and ß-carotene were not significantly associated with colorectal cancer risk.

**Conclusions:**

Further prospective studies are needed to confirm the contrasting effects of high total antioxidant intake on risk of colon and rectal cancers.

## Introduction

In 2012, colorectal cancer was the third most common cancer worldwide in men, with about 746,000 cases diagnosed, and the second most common cancer in women with about 614,000 cases diagnosed [1]. Around 55% of cases occur in developed countries [1]. The panel of the World Cancer Research Fund/American Institute for Cancer Research (WCRF/AICR) [2] reported that there is ‘convincing’ evidence that foods high in dietary fiber protect against colorectal cancer, and that consumption of red meat, processed meat and alcohol (especially in men) increase the risk of developing colorectal cancer. The panel found ‘limited’ evidence that non-starchy vegetables, fruits, and foods containing vitamin D, protect against colorectal cancer, and that cheese, foods containing sugar, animal fats and iron increase colorectal cancer risk.

Nevertheless other studies and meta-analyses have found that a diet rich in fruit and vegetables reduces the risk of several common cancers, particularly those of the digestive tract [3, 4].

Foods of plant origin contain various antioxidants [5, 6]. Those with high antioxidant content include coffee, chocolate, berries, vegetables of the brassica family, red wine, and wholegrain cereals [7–9]; and these have been proposed as protective against cancer, in part because antioxidants counteract free radicals in the body [10, 11]. Free radicals can trigger carcinogenesis by permanently damaging cellular components, particularly DNA [12, 13]. There is also evidence that dietary antioxidants can protect against cancer by other mechanisms [14]. However, the studies that investigated a proposed protective effect of antioxidants against colorectal cancer reported null [15] or limited [16] results.

As different antioxidants in the diet may act synergistically or at least additively to prevent cancer [14], it seems important to assess overall antioxidant intake when investigating the effects of diet on cancer risk. However, studies that investigated the total antioxidant capacity (TAC) of the diet reported no relation [17] or an inverse relation [18] between TAC and the development of colorectal cancer. We therefore set out to investigate whether dietary TAC intake was associated with the risk of developing colorectal cancer in participants of the Italian section of the European Prospective Investigation into Cancer and Nutrition (EPIC) study. We also assessed whether dietary vitamin E, vitamin C and ß-carotene influenced colorectal cancer risk.

## Materials and Methods

### Participants

A total of 47,749 volunteers (15,171 men and 32,578 women) were recruited to Italian EPIC, mainly between 1993 and 1998. EPIC is a prospective cohort study investigating the role of biological, dietary, lifestyle and environmental factors in the etiology of cancer and other chronic diseases. Volunteers were recruited from five centers: Varese and Turin (Northern Italy), Florence (Central Italy), Naples and Ragusa (Southern Italy). Only women were recruited in Naples. The ethics committee of the International Agency for Research on Cancer, Lyon, France, and the Cancer Research and Prevention Institute (ISPO), Florence, Italy, approved the EPIC Italy study protocol. The study complies with the Helsinki declaration, and participants gave informed consent to use clinical data for research.

In the present study, we examined 45,194 participants (14,172 men and 31,022 women) who completed a dietary questionnaire at enrolment, excluding those: with cancer at recruitment (except non-melanoma skin cancer); lost to follow-up soon after baseline; with missing information on anthropometry, lifestyle or education; with ratio of total energy intake (determined from the questionnaire) to basal metabolic rate [19] at either extreme of the distribution (cut-offs first and last half-percentiles) (n = 1,320); and with implausible coffee consumption (>15 cups/day; n = 16).

#### Dietary information

Diet was assessed using validated [20] semi-quantitative food frequency questionnaires (FFQs) designed to capture eating behavior over the previous year in different regions of Italy. One FFQ was used for the centers of Varese, Turin and Florence, another for Ragusa, and another for Naples. The FFQs contained questions about 188, 217 and 140 food items, respectively [21]. Macro- and micro-nutrient values for each food item were extracted from the FFQs using the software Nutrition Analysis of Food Frequency Questionnaire, developed by the Epidemiology Unit of the National Cancer Institute of Milan [21]. This software converts questionnaire responses into frequencies of consumption and average daily quantities of foods, energy and nutrients making use of the Italian Food Composition Tables [22]. In order to compare the different questionnaires in terms of similar but unequal food items, the program has flexible classification system that can group similar food items together according to the presence of a given component [21]. Repeated open-ended interviews, conducted over a year and investigating food consumed over the preceding 24 hours were used to validate the FFQs [20].

#### Determination of TAC

Food items were analyzed for TAC using the Trolox Equivalent Antioxidant Capacity (TEAC) assay [8;23]. The TEAC method employs the radical cation 2,2'-azinobis-(3-ethylbenzothiazoline-6-sulfonic acid) (ABTS•+) which is generated by oxidation of ABTS with potassium persulfate and is reduced by a wide range of hydrogen-donating antioxidants. Addition of diluted food extracts to a solution of the blue/green ABTS•+ chromophore reduces it to colorless ABTS, to an extent and on a time-scale depending on the activity and concentration of antioxidants present [24]. The ability of food extracts to reduce the chromophore was compared to that of Trolox, a water-soluble vitamin E analog used as a standard: thus TAC values are expressed as mmol Trolox equivalent/day. The TEAC method is better able to measure the contributions of both hydrophilic and lipophilic antioxidants to TAC than other methods [8, 23, 24].

TAC values (either pre-exiting or specifically determined for this study) for individual foods were added to the Food Composition Tables. The Nutrition Analysis of Food Frequency Questionnaires software used the TAC values, now in the Food Composition Tables, to convert the food quantities recorded in the FFQs into average daily TAC values for each person.

#### Lifestyle

Participants completed a standardized lifestyle questionnaire, designed to obtain detailed information on reproductive and medical history, physical activity, alcohol consumption, smoking, exposure to environmental tobacco smoke, education, and other socioeconomic variables.

#### Anthropometry

Weight and height were measured at enrollment using a standard protocol [25], with participants wearing light clothes, without shoes. Body mass index (BMI) was calculated as kg/m^2^.

### Identification of colorectal cancer cases and follow-up

In Varese, Turin, Florence and Ragusa, incident colorectal cancer cases were identified by linkage of the study database to the databases of the regional cancer registries, which are considered high quality registries with nearly complete cancer registration [26]. In Naples, incident cases were identified through linkage to the regional archive of hospital discharges, and by direct telephone contact where necessary.

Colon cancers were primary cases identified by codes C18.0-C18.9 of the International Statistical Classification of Diseases, 10th Revision. Rectal cancers were identified by codes C19 (rectosigmoid junction) and C20 (rectum). Anal cancers were excluded.

Follow-up ended December 31 2006 in Florence, Varese and Naples; and December 31 2008 in Ragusa and Turin. This difference was due to the fact that updated cancer registry and hospital discharge file availability varied.

### Statistical methods

Person-time of follow-up for each participant was calculated from the date of enrolment to the date of cancer diagnosis, death, loss to follow-up, or end of follow-up, whichever occurred first. Cox proportional hazard models were used to assess the association of cancers with TAC, vitamin E, vitamin C, and ß-carotene, all categorized into tertiles of intake, defined on the whole cohort, with lowest tertile as reference. The proportional hazards assumption for TAC, vitamin E, vitamin C, β-carotene, and all other covariates in relation to breast cancer risk was tested using the Grambsch and Therneau method [27]. In all cases, the proportional hazards assumption was satisfied.

We present three models: model 1 adjusted only for age and sex; model 2 additionally adjusted for BMI (continuous), height, smoking status, total physical activity (inactive, moderately inactive, moderately active and active) and education (≤8 years, >8 years); and model 3 additionally adjusted for intakes of: alcohol (≤0,1; >0.1- ≤ 12; >12- ≤ 24; >24 g/day), non-alcohol energy, calcium, red meat, processed meat, and dietary fiber (all continuous). All models were stratified by center (separate baseline hazard functions for each center within the Cox model). Vitamin C and E were expressed in mg/day, and ß-carotene in μg/day. Analyses were carried out for all types of colorectal cancer and for colon and rectal cancer separately.

Linearity of trends across categories was tested treating median values of each tertile as a continuous variable in the Cox model. P values for interaction between sex and TAC were estimated adding to the model a product term of sex and TAC tertiles. P values <0.05 were considered significant. Stata version 11.2 (College Station, TX) was used to perform the analyses.

## Results

Four hundred thirty-four colorectal cancers (325 colon cancer, 109 rectal cancer) were identified during a mean follow-up of 11.28 years. Varese had the highest incidence of colorectal cancer among males (32 cases per 23,292 person-years), followed by Turin (89/71,298 person-years), Florence (36/35,525 person-years) and Ragusa (24/31,313 person-years). Florence had the highest incidence of colorectal cancer among females (91/103,419 person-years), followed by Turin (43/51,940 person-years), Varese (75/101,648 person-years), Ragusa (17/34,098 person-years) and Naples (female only 27/57,078 person-years).

Table 1 shows baseline characteristics of participants by tertiles of dietary TAC. Mean age decreased from lowest to highest tertile, while mean BMI, height and non-alcohol energy intake increased with increasing TAC tertiles. Participants with highest TAC were more active, better educated, and smoked more than those in lower tertiles. Participants from Turin had higher dietary TAC than the other centers. The main contributors to dietary TAC intake were coffee (42.4%), fruit (16.6%), wine (15.6%), and bread (4.5%). Other minor contributors were chocolate, vegetables, soups, tea, and fruit juices (data not shown).

**Table 1:** Baseline characteristics of study participants by tertile of dietary TAC intake.

Characteristic	TAC 1st tertile (≤5.26 mmol/day)	TAC 2nd tertile (5.27–7.22 mmol/day)	TAC 3rd tertile (≥7.23 mmol/day)
Mean age (SD) years	51.4 (8.2)	50.4 (7.8)	49.8 (7.6)
BMI (SD) kg/m^2^	25.8 (4.3)	25.9 (4.1)	26.2 (3.8)
Non-alcohol energy, (SD) kcal/day	1956.2 (547.8)	2274.5 (575.0)	2730.2 (702.4)
Height (SD) cm	160.5 (8.2)	162.0 (8.5)	165.3 (9.0)
Physical activity (%)			
*Inactive*	40.1	34.5	25.4
*Moderately inactive*	34.0	34.2	31.8
*Active*	24.0	29.7	46.3
Centre (%)			
*Turin*	28.9	30.3	40.8
*Varese*	34.6	34.1	31.3
*Florence*	33.3	32.7	34.0
*Naples*	37.8	37.5	24.7
*Ragusa*	34.3	34.7	31.0
Gender (%)			
*Male*	20.6	29.2	50.2
*Female*	39.2	35.2	25.6
Smoking status (%)			
*Current smoker*	23.7	33.0	43.3
*Ex-smoker*	28.3	33.1	38.6
*Never smoked*	41.8	33.8	24.4
Education *>8 y (%)*	31.8	34.1	34.1

No significant association between dietary TAC and colorectal cancer as a whole was found. However, in model 3 (fully-adjusted), high TAC intake was significantly associated with reduced risk of colon cancer (hazard ratio (HR): 0.63; 95% confidence interval (CI): 0.44–0.89, highest tertile vs. lowest, P trend: 0.008). By contrast, increasing TAC intake was associated with significantly increasing (P trend 0.007, fully-adjusted model) risk of rectal cancer (2nd tertile HR: 2.09; 95%CI: 1.19–3.66; 3rd tertile 2.48 95%CI: 1.32–4.66) (Table 2).

**Table 2:** HRs for developing colorectal, colon and rectal cancer by increasing tertiles of dietary TAC.

	TAC 1st tertile	TAC 2nd tertile	TAC 3rd tertile	P trend^5^
**Range** ^**1**^ **:**	≤5.26	5.27–7.22	≥7.23	
**Colorectal cancer**				
Cases:	134	154	146	
Person-years:	168,997	169,956	170,659	
**Model 1 HR** ^**2**^ **(95%CI):**	1	1.20 (0.95–1.52)	1.07 (0.84–1.37)	0.649
**Model 2 HR** ^**3**^ **(95%CI):**	1	1.19 (0.94–1.50)	1.05(0.82–1.35)	0.774
**Model 3 HR** ^**4**^ **(95%CI)**	1	1.12 (0.88–1.43)	0.88 (0.65–1.19)	0.353
**Colon cancer**				
Cases	114	115	96	
Person-years	168,878	169,700	170,321	
**Model 1 HR** ^**2**^ **(95%CI):**	1	1.07 (0.82–1.39)	0.85 (0.64–1.12)	0.236
**Model 2 HR** ^**3**^ **(95%CI):**	1	1.06 (0.81–1.37)	0.84(0.63–1.12)	0.228
**Model 3 HR** ^**4**^ **(95%CI)**	1	0.96 (0.73–1.26)	**0.63 (0.44–0.89)**	**0.008**
**Rectal cancer**				
Cases:	20	39	50	
Person-years:	168,215	169,138	170,055	
**Model 1 HR** ^**2**^ **(95%CI):**	1	**1.98 (1.15–3.41)**	**2.32 (1.35–3.97)**	**0.003**
**Model 2 HR** ^**3**^ **(95%CI):**	1	**1.94 (1.13–3.34)**	**2.18(1.26–3.76)**	**0.008**
**Model 3 HR** ^**4**^ **(95%CI)**	1	**2.09 (1.19–3.66)**	**2.48(1.32–4.66)**	**0.007**

^1^ mmol Trolox equivalents/day.

^2^ Adjusted for age and sex; stratified for center.

^3^ Additionally adjusted for BMI, height, smoking status, education and total physical activity; stratified for center.

^4^ Additionally adjusted for intakes of alcohol, non-alcohol energy intake, red meat, processed meat, calcium and dietary fiber; stratified for center.

^5^P trends were calculated from the Cox model treating each category as a continuous variable.

Vitamin C had no significant association with colorectal, colon, or rectal cancer (Table 3). ß-carotene was not significantly associated with colorectal or rectal cancer, but the risk of colon cancer in the second tertile was significantly greater than reference (HR: 1.33; 95%CI 1.01–1.75), with no increased risk in the third tertile (P trend 0.306). Vitamin E was not significantly associated with colorectal or colon cancer. However rectal cancer risk in the second tertile of vitamin E intake was significantly greater than reference (HR: 1.75, 95%CI: 1.05–2.90), with no increased risk in the third tertile (P trend 0.431). There was no interaction between sex and TAC (data not shown).

**Table 3:** HRs for developing colorectal cancer, colon and rectal cancer by increasing tertiles of vitamin C, ß-carotene and vitamin E intake.

Antiox. intake (tertiles)	Daily intake^2^	Colorectal cancer		Colon cancer		Rectal cancer	
		Cases/ person-year	HR^3^(95%CI)	Cases/ person-year	HR^3^(95%CI)	Cases/ person-year	HR^3^(95%CI)
**Vitamin C, mg/d**							
**1**	83.20	158/168,601	1	125/168,417	1	33/167,781	1
**2**	131.39	159/170,192	1.06 (0.85–1.35)	114/169,862	0.97 (0.74–1.26)	45/169,429	1.46(0.91–2.35)
**3**	200.96	117/170,819	0.83(0.61–1.11)	86/170,619.	0.75 (0.54–1.06)	31/170,199	1.10 (0.60–2.01)
**P trend** ^**1**^			0.190		0.098		0.837
**ß-carotene, μg/d**							
**1**	1822	149/167,558	1	107/167,287	1	42/166,888	1
**2**	2944	159/169,974	1.15(0.91–1.45)	130/169,783	**1.33 (1.01–1.75)**	29/169,058	0.70 (0.43–1.16)
**3**	4776	126/ 172,080	0.91(0.69–1.20)	88/171,827	0.90 (0.65–1.25)	38/171,462	0.92 (0.54–1.57)
**P trend** ^**1**^			0.372		0.306		0.980
**Vitamin E, mg/d**							
**1**	5.82	159/168,344	1	129/168,179	1	30/167,528	1
**2**	8.04	154/170,110	1.04 (0.82–1.33)	108/169,777	0.89 (0.67–1.18)	46/169,346	**1.75 (1.05–2.90)**
**3**	10.96	121/171,158	0.83(0.60–1.18)	88/170,942	0.72 (0.49–1.06)	33/170,534	1.36 (0.69–2.68)
**P trend** ^**1**^			0.284		0.089		0.431

^1^P trend based on Cox model treating each category as a continuous variable.

^2^ Values are medians.

^3^ Adjusted for age, sex, BMI, height, smoking status, education, intakes of alcohol, total physical activity, fiber, non alcohol energy intake, red meat, processed meat, calcium and dietary fiber; stratified for center.

## Discussion

We found no association between dietary TAC and overall risk of colorectal cancer. However, when colon and rectal cancers were considered separately, increasing TAC intake was associated with reducing risk of colon cancer and increasing risk of rectal cancer, both with significant trends. Intake of individual antioxidants (vitamin C, vitamin E and ß-carotene) was not significantly associated with colorectal cancer risk, in agreement with the 2007 WCRF/AICR panel report and recent reviews [15, 16, 28]. Thus, our evaluation of the total antioxidant capacity of the diet has found effects not revealed by investigation of individual antioxidants.

To our knowledge only two previous studies have assessed the effect of dietary TAC on colorectal cancer risk. A prospective study on US male health professionals found that high TAC intake from coffee, decaffeinated coffee, and tea was associated with reduced rectal cancer risk [18]. However the effect was not present for total TAC intake, and the authors suggested that antioxidants may not be driving the association, and that other types of compounds present in tea and coffee might be responsible for the anti-rectal cancer observation [18]. An Italian multicentric case-control study found a significant inverse association between TAC and colorectal cancer risk, which appeared somewhat stronger for rectal cancer than for colon cancer [17].

Our finding that increasing TAC intake was significantly associated with increasing risk of rectal cancer is in contrast to the findings of both the US and Italian studies [17, 18] while our finding that colon cancer risk was significantly reduced in those with highest TAC intakes is in line with the findings of the Italian study [17].

We expected that high dietary TAC would be protective against both colon and rectal cancer, and our finding that it is associated with increased risk of rectal cancer requires careful examination. It is known that etiologic factors for the two cancers differ [29–32]; that colon and rectum derive from different segments of embryonic intestinal tract, and differ in function, pH, and exposure to fecal matter [29, 30]; furthermore levels of several bacterial hydrolytic and reductive enzymes involved in the production of mutagenic metabolites vary regionally within the large bowel [33]. Any of these differences might contribute to contrasting effects of dietary TAC.

Furthermore, while antioxidants are generally considered to have beneficial effects in the body because of their ability to reduce oxidative stress, in some conditions they or their metabolites may have pro-oxidant effects [34, 35]. In particular the polyphenols widely present in human diet may be pro-oxidant in high doses or in the presence of iron or copper ions, and may therefore cause oxidative damage to certain tissues and hence initiate carcinogenesis [13, 36, 37].

In our study we estimated that TAC was mainly derived from coffee (42%), fruit (17%), and wine (16%). As regards the TAC of coffee, this is mainly due to phenolic compounds and also to melanoidins formed by the Maillard reaction during roasting [9, 38]. Chlorogenic acids are major phenolic constituents of coffee, only 30% of which appear to be absorbed by the small intestine [39]. The remaining proportion arrives in the colon where it may be metabolized or absorbed (approximately 21% of total). Chlorogenic acids and their metabolites not absorbed by the colon, reach the rectum where they may exert various effects, perhaps differing from those in the colon [39].

The NIH-AARP Diet and Health Study found that coffee consumption was inversely associated with colon cancer [40]. A recent meta-analysis [41] also found that, among case-control studies, coffee consumption was significantly related to lowered risk of colorectal cancer and colon cancer, but not rectal cancer. Both these findings are consistent with our findings, since TAC was mainly derived from coffee in our study.

Our finding that rectal cancer risk increased with antioxidant intake is also consistent with the results of a large prospective cohort study which investigated relationships between colorectal cancer risk and intakes of total fluid and specific beverages. This study found that coffee intake was associated with increased risk of rectal cancer in men. The authors suggested that this might be due to the presence of carcinogens (possibly acrylamide) in coffee [42]. Finally, the large prospective EPIC study, which analyzed data on 477,071 participants from centers across Europe (and included EPIC Italy data) found that high coffee consumption was non-significantly associated with increased rectal cancer risk (HR 1.20, 95% CI 1.00–1.44; p trend 0.15) [43].

As regards TAC from fruit: vitamin C, vitamin E, carotenoids and polyphenols are the main contributors to antioxidant capacity from this source [9, 44]. The absorption of these micronutrients is influenced by several factors (e.g. the absorption of fat-soluble forms is facilitated by dietary fat and inhibited by dietary fiber) [44] and their concentrations in body tissues may vary widely, with biological action also varying with concentration [44]. We found no association between intakes of vitamin C, vitamin E and ß-carotene and colorectal cancer incidence. Similar results were also reported by the WCRF/AICR that found only limited evidence of reduced colorectal cancer risk associated with high fruit consumption [2, 16].

As regards TAC from wine, red wine is rich in polyphenols [9], including quercetin, which has been shown to inhibit the growth of colon cancer and other cancer cells [45]. The antioxidant polyphenol resveratrol is present in wine and may contribute to preventing colon cancer [46]. However alcohol intake is clearly associated with increased risk of colorectal cancer [47]. The risk is greater for rectal cancer than cancers of distal and proximal colon [47]. The greater rectal cancer risk could be due to greater epithelial hyper-regeneration in rectum [48] and higher concentration of (mutagenic and carcinogenic) acetaldehyde in rectum than colon [49]. In our study the relationship between high TAC intake and risk of rectal cancer was maintained after adjusting for alcohol intake, hence the mechanism underlying the adverse effect of dietary TAC on rectal cancer is unclear.

Strengths of our study are its prospective design, large sample size, long follow-up (15 years), small number of participants lost to follow-up, and the fact that validated and reproducible FFQs were used to assess diet and dietary TAC [50]. However TAC intake was only assessed at baseline and may not reflect TAC intake over a long period. Furthermore we cannot rule out confounding by factors we were unable to estimate or estimated sub-optimally our questionnaires (FFQ and lifestyle). Furthermore relatively few cases of rectal cancer were diagnosed, so our finding that high TAC was associated with increased the risk of this cancer requires further investigation.

To conclude, our findings suggest that a high TAC diet may play a role in reducing risk of colon cancer, but not rectal cancer, for which increased risk with increasing TAC was found. Further prospective studies are needed to confirm the contrasting effects of total antioxidant intake on risk of colon and rectal cancers.

## Abbreviations

ABTS 2,2’: azino-bis(3-ethylbenzothiazoline-6-sulphonic acid)
AICR: American Institute for Cancer Research
BMI: body mass index
CI: confidence interva
EPIC: European Prospective Investigation into Cancer and Nutrition
FFQ: food frequency questionnaire
HR: hazard ratio
SD: standard deviation
TAC: total antioxidant capacity
TEAC: trolox equivalent antioxidant capacity
WCRF: World Cancer Research Fund
